# A qualitative study of health professionals’ uptake and perceptions of malaria rapid diagnostic tests in Burkina Faso

**DOI:** 10.1186/s12936-016-1241-6

**Published:** 2016-04-06

**Authors:** Sylvie Zongo, Valérie Farquet, Valéry Ridde

**Affiliations:** Département Socio-Économie et Anthropologie du Développement, Institut des Sciences des Sociétés (INSS-CNRST), 03 BP 7047 Ouagadougou, Burkina Faso; University of Montreal Public Health Research Institute (IRSPUM), 7101 Avenue du Parc, Montreal, QC H3N 1X9 Canada

**Keywords:** Malaria, Rapid diagnostic tests, Health professionals’ perceptions, Artemisinin-based combination therapy, Burkina Faso

## Abstract

**Background:**

Since 2012, rapid diagnostic tests (RDT) for malaria have been in use nationwide in Burkina Faso. The objective is to strengthen health professionals’ diagnostic capabilities and promote good therapeutic practices. A qualitative study was conducted to learn about the adoption of this tool in the natural context of a national scale-up policy.

**Methods:**

This study involved five health centres in two health districts. Twenty-eight individual interviews were conducted in 2013 with health professionals and members of the health district management teams. Health professionals’ RDT use and drug prescription practices were observed during 278 curative care consultations over 5 weeks.

**Results:**

Health professionals assessed the use of RDT positively as it allowed them to reach clear and accurate diagnoses and above all to deliver appropriate, rational care. However, the introduction of RDTs did not really change their diagnostic practices or prescribing practices for artemisinin-based combination therapy (ACT). They continued to rely predominantly on symptoms in establishing their diagnoses because of doubts regarding the reliability of the tests and the occasional stockouts of RDTs experienced by the health centres. Patients with negative RDT results continued to receive anti-malarial treatments. However, the situation remains quite heterogeneous.

**Conclusion:**

The use of RDTs points to the co-existence of official standards and different standards applied in practice. Setting up regular supervision activities provided an opportunity to observe and understand the various obstacles encountered by health professionals and to monitor how official directives are put into practice. For efficient use of RDTs and their results, health professionals need information and directives that are up-to-date and standardized.

## Background

The World Health Organization (WHO) estimated there were 216 million malaria episodes in 2010 [1]. In Burkina Faso, around eight million cases of malaria are reported each year. Over the past 5 years, malaria has been the primary reason for consultation, hospitalization, and death in health centres, particularly among children under the age of 5 years [2]. Recent health statistics show 4166 deaths reported for this age range, for a 2 % lethality [3]. Rapid diagnoses and appropriate drug treatment are now available. This drug treatment, which uses artemisinin-based combination therapy (ACT), should be administered only after evidence of the pathogen has been obtained through biological testing [1]. However, given the scarcity of microscopes and of qualified staff to perform biological testing on blood samples in basic health services centres, since 2004 WHO has recommended the use of antigen-detecting rapid diagnostic tests (RDTs). These are biological tests that can rapidly (within 10–15 min) detect the antigen for malaria parasites from micro-samples obtained by finger-prick and applied to a test strip [4].

In Burkina Faso, biological testing for malaria treatment is done using two basic techniques, either microscopic diagnosis using thick blood smears or RDT. Official directives stipulate that all suspected cases of malaria must be confirmed by one of these techniques before treatment [5]. RDTs are used primarily to confirm a malaria diagnosis in primary care centres that do not have microscopes. They were introduced, beginning in 2009, in 29 health districts of six pilot regions for use in persons over the age of 5 years; Their introduction was preceded in 2008 by a series of top-down training sessions provided first at the regional level, then at the health district level, and finally at the health centre level, represented by the clinical managers. As of 2010, RDTs are used for patients of all ages, and training sessions have been organized in all regions following the same top-down approach [6]. An evaluation, conducted in six primary care centres, of the use of RDTs in this controlled pilot context showed that health professionals did not use RDTs when these were required, preferring to maintain their usual clinical diagnostic routines and treatment algorithms [7]. When RDTs were used, the majority of negative results were ignored by the health professionals, who continued to prescribe ACT. These practices in Burkina Faso were associated with a cautious attitude toward RDTs [6]. Studies conducted elsewhere in Africa have shown that health professionals consider the use of RDTs to be an important step in obtaining appropriate care and a means of determining the etiology of fever cases, helping to strengthen diagnostic capacities and improve the quality of therapeutic indications [8]. However, health professionals’ use of RDTs continues to be problematic, especially with regard to managing results. Studies have revealed that health professionals’ prescriptions are not always based on RDT results [9, 10]. This situation has resulted in continued inappropriate use of anti-malarials, which the introduction of RDTs was intended to curtail [11]. Health professionals’ compliance with directives regarding diagnosis and treatment is a fundamental prerequisite for effective implementation of malaria management policies [1].

Since 2010, RDTs have been used throughout Burkina Faso for all patients [12]. This national scale-up of RDTs in a natural context of public policy calls for reflection on the adoption of this tool, how it is perceived by health professionals, and its impact on care organization and health professionals’ practices. To ensure the sustainability of RDTs, it is important to understand the logic driving health professionals’ adoption of them [13, 14]. This is even more the case when considering that, up to now, the available knowledge on RDT use has been derived only from pilot projects and relatively controlled situations. As such, there is a real need for knowledge drawn from a natural context [15, 16]. The objective of this study was to understand health professionals’ adoption, perceptions and use of RDTs in the natural context of a national scale-up of a public policy.

## Methods

### Study setting

The study was conducted in the health districts of Kaya and Zorgho, in Burkina Faso, where research is engaged on the national anti-malaria programme [17]. These two sites were selected for purposes of this evaluation because they are comparable in terms of rainfall, income levels, population composition, and health professionals’ presence and qualifications. Kaya health district is situated in the Centre-North region, 90 km northeast of the capital, Ouagadougou. It has 564,867 inhabitants, 56 % of whom live within a 5-km radius of a primary health care centre. In 2014, the district reported 210,775 cases of uncomplicated malaria, including 127,151 cases in children under five, 6098 severe cases, and 57 deaths, for a 0.9 % lethality. Zorgho health district is situated in the Central Plateau region, 203 kms east of Ouagadougou. It has a population of 393,778, of whom 49.3 % live within a 5-km radius of a primary health care centre. The incidence of malaria is higher in Zorgho than in Kaya. In 2014, there were 204,775 uncomplicated cases of malaria reported, including 99,662 cases in children under five, 10,447 severe cases, and 109 malaria-related deaths, i.e., a lethality of 1.0 %, compared to a national average of 1.2 % [3]. The organization of healthcare is similar in both districts. User fees are collected for consultations and treatments in both districts, except for children under 5 years (0–59 months) in Kaya, who have been exempted from direct user fees and ambulance transportation costs since July 2011 [18].

### Methodological approach

In the two health districts five primary care health and social promotion centres (CSPS) were selected, of which three were in urban areas and two in rural areas. The selection was aimed at providing a diversity of situations in the two districts and to enable urban–rural comparisons. The field survey was conducted between 19 February and 28 March, 2013. An exploratory visit was conducted in December 2012 in Kaya to learn more about the setting and the status of RDT use, present the study to the clinical teams, and pre-test the data collection tools.

Two qualitative data collection tools were used: individual interviews (n = 24) and observations. The individual interviews began with 20 health professionals. In rural health centres, the number of health professionals varied from two to three. Their statuses were different but they all performed the same acts (including RDTs). Given their limited numbers, it was possible to interview all of them. In the urban centres, where there were many more health professionals, the decision was taken to interview only those working in clinics, as that was where curative care consultations occurred. Only those health professionals who were willing to talk with the researchers were interviewed. Nevertheless, every effort was made to obtain a diverse sample of health professionals, in terms not only of their status and function in the health centres, but also of their participation in RDT training sessions. The interviews explored their knowledge about RDTs, what use they made of them, their prescribing practices in relation to the test results, their perceptions of the tool and of how it was received by patients, especially mothers of children under five.

In addition, interviews were conducted with four members of the two districts’ management teams (ECD), including the district medical officers (MCD) or their assistants, the managers of the district distribution depots (DRD) who look after RDT stocks, and the managers of the health information and epidemiological surveillance centres (CISSE). A number of curative care consultations were also observed to understand the procedures for medical care of the ill, particularly in cases of fever, and especially the procedures for determining whether or not to use RDTs. In all the CSPSs, a total of 278 curative care consultations in clinics were observed. To conduct these observations, between 4 and 10 days were spent in each of the different CSPSs.

All interviews were audio-recorded and fully transcribed into a word-processing program. The transcripts underwent content analysis by site and by topics addressed in the interview guides. Added to these were the four interviews (three health professionals and one ECD member) conducted during the exploratory phase, which were recorded using written notes, bringing to 28 the total number of interviews analysed. Tables 1, 2 summarize the data collection process.

**Table 1:** Summary of the data collection process

Sites	Number of consultations	Number of days	Number of interviews
CSPS-urban A (Kaya)	90	09	07
CSPS-urban B (Kaya)	112	10	06
CSPS-rural (Kaya)	24	04	03
CSPS-urban (Zorgho)	32	05	05
CSPS-rural (Zorgho)	20	05	02
ECD (Zorgho)			02
ECD (Kaya)			03
Total	278	32	28

**Table 2:** Participants’ status and sociodemographic characteristics

Sites	Gender		Status					
	Female	Male	AIS	IB	IDE	MCD/assistant	DRD	CISSE
CSPS urban A (Kaya)	06	01	01	02	04	00	00	00
CSPS urban B (Kaya)	03	03	02	00	04	00	00	00
CSPS rural (Kaya)	02	01	02	01	00	00	00	00
CSPS urban (Zorgho)	01	04	02	01	02	00	00	00
CSPS rural (Zorgho)	00	02	01	01	00	00	00	00
ECD (Kaya)	01	02	00	00	00	02	00	01
ECD (Zorgho)	01	01	00	00	00	01	01	00
Total	14	14	08	05	10	03	01	01

## Ethical considerations

The study received ethical approval from the research ethics committees of Burkina Faso and the University of Montreal Hospital Research Centre (CRCHUM). The results of the analyses were presented to the key stakeholders at a workshop held in November 2013.

## Results

### Relatively informal training in the use of RDT

As malaria is the predominant reason for patient visits, clinicians devote considerable time to its diagnosis. According to the clinicians, before the introduction of RDTs, diagnosis was based essentially on the patient’s symptoms. Most cases of fever and symptoms suggestive of malaria were regarded as malaria. Anti-malarials were immediately prescribed, even in cases where the symptoms might suggest other pathologies. The diagnosis was presumptive and anti-malarials were prescribed according to a cautionary logic of making sure the patient was covered.

*“Before RDTs, there was a lot of investigating, and in the malaria programme there was a dictum to the effect that ‘whenever there’s fever, treat for malaria first,’ so if someone came in with fever, even though we would still investigate the possibility of other things, nevertheless the first line of treatment included an anti*-*malarial. Even if there’s no scientific evidence there, if it’s based on what the mother or father says, or what the health worker observed, it’s certainly true that, even if he says there’s coughing, that can cause fever, but when there’s fever, we treat for malaria and maybe also pneumonia… And even if that’s not the right diagnosis, to cast a wide net we prefer to include [an anti*-*malarial].”* State certified nurse (IDE), male, urban CSPS, Zorgho.

RDTs were introduced following a training process that began with health centre managers. It was carried out using different methods: health professionals received “formal” training, either from the Ministry of Health or from organizations working in their health district.

In Zorgho health district, the use of RDTs was preceded in 2011 by an information workshop for CSPS managers.

In Kaya health district, the scale-up in 2012 was preceded by training on RDT use, provided from April 23–28 to health professionals who were, in turn, mandated to train their colleagues in the CSPSs.

*“The scale*-*up in the district was done in 2012. There was training provided from April 23 to 28, I believe…They trained two health professionals per CSPS. It believes it was the malaria programme. In any case, the implementation was done after the training, around June, I think, and then each CSPS started when it wanted. They didn’t all start at the same time.”* District management team (ECD), female, Kaya.

Before this, the NGO Save the Children, as part of its malnutrition management programme, had provided some of the Kaya CSPSs with RDTs starting in 2010, for managing children under five, and in that initiative had trained some of the CSPS managers in the use of this tool.

The reasoning behind the training provided for the scale-up was that the knowledge acquired would subsequently be transferred to personnel in the health centres who had not attended the training sessions. In both districts, RDTs were introduced into the CSPSs by either the centre managers or their representatives who had undergone training. Those who were trained were expected, in turn, to train their colleagues who had not attended the session. For most health professionals, their first contact with RDTs and their knowledge acquisition occurred in their own health centre through transfer activities (verbal, practices, and/or posting of national directives) carried out by those who had been trained. In both districts, this was the process by which the majority of health professionals encountered in the study had learned to use RDTs. Transfer activities took several forms, including unit meetings that focused specifically on RDTs or in which information on RDTs was included among other items. For other health professionals, the learning was transferred through practice, that is, during consultations with patients.

According to the health professionals, these different methods of teaching RDT use enabled them to perform the test easily but resulted in differences, and even divergences, in how the tool was adopted. These were seen not only in the health professionals’ knowledge, which was inconsistent, but also in their practices that did not necessarily follow official directives.

While the health professionals were more or less knowledgeable about the objectives for the use of the tool, its actual manipulation was the subject of much discussion and a variety of practices. For example, there were diverse opinions regarding the amount of buffer solution to use and especially the wait time for results. According to one health professional:

*“Just three drops. If the blood is very sticky, you can add a drop because sometimes it congeals. If the blood is sticky, it’s not flowing fast, we can put four drops to dilute the blood even more, it might not work. From what I know, it’s three drops; if it’s four drops, too, then I’m waiting for someone to let me know that…. We can have the results in 10 to 15* *min.”* Health outreach worker (AIS), female, urban CSPS A, Kaya.

In reality, there were different opinions. The number of drops varied from one professional to another, and even for the same professional, from one consultation to another. Likewise, with regard to the wait time for results, observations of curative consultations on all the sites revealed that an average of five to 10 min was spent with each patient, even when an RDT was performed. Yet according to official directives (posted in some consultation rooms), they were supposed to wait at least 15 min before reading and interpreting the results—a time that many health professionals considered overly long. One student nurse at the urban Zorgho CSPS explained what happened during curative consultations carried out under the supervision of a certified health professional:

*“In principle, it’s 15* *min, but if you had to wait that long*—*well! It’s just that, right now, there’s no one here, but otherwise there are times when the bench (of the waiting room) is filled to right out the door. If you had to spend 15* *min with a patient, oh boy! The patients themselves would come pounce on you here!”* Student nurse, female, urban CSPS, Zorgho.

One consequence of these coping strategies is that some patients are mistakenly diagnosed, as was observed in one of the urban CSPSs in Kaya. In a consultation for a 10-month-old child presenting with “hot body and cough with vomiting,” which the mother had treated with paracetamol, a nurse in the urban CSPS A of Kaya decided to perform an RDT. Five minutes later, without the nurse having observed that the control line indicated a negative RDT, she nevertheless reached a diagnosis: “uncomplicated malaria + pneumonia” and prescribed ACT syrup and an antibiotic as treatment. After the mother had left, as the student assisting the nurse was about to dispose of the test in the trash, she noticed that the second line, showing that the test was positive, had appeared, although faintly. When she informed the nurse, “*Look, it’s positive, there’s a faint line, and the woman has already left*,” the nurse replied, “*Oh, well, since there was an anti*-*malarial in there, it’s okay!*”

Most of the health professionals in the two districts had learned to use RDTs from transfer activities conducted by others who had been trained. In Zorgho district, the directives on RDT use, posted in the consultation rooms, were also a source of information. This posting of the directives for performing RDTs and of the decision algorithm for suspected cases of malaria was an element that differentiated the health centres in the two districts. In the health centres investigated in Zorgho (formally or informally), these two postings were present in the consultation rooms and were sometimes accompanied by handwritten notes on the use of RDTs. Health professionals referred to them regularly to explain certain points during the interviews to show either what they did, or what they were supposed to do (for example, for questions on the use of the tool and on prescribing practices based on RDT results).

### The same tests performed by health professionals with different status

The use of RDTs was not limited to a single category of health professional. Everyone who saw patients in consultation performed the tests, regardless of their status. However, it was how care was organized that determined who was involved. It was observed that those who conducted the consultations were generally the ones who performed the RDTs. In health centres with staffing shortages, a professional might be working alone. This was the case most often in afternoons or during on-call shifts. Based on the principle that anyone who consults should be able to perform RDTs, it becomes difficult to prohibit anyone from doing it, or to limit it to only one category of health professional. Thus, anyone who received patients in consultation then performed the tests, regardless of their status.

*“In principle, according to the Ministry of Health, AISs shouldn’t be doing them, but on the ground, the CSPSs have staff shortages and so they have to involve the AISs.”* ECD, male, Kaya.

*“Everyone does them. In any case, everyone who carries out consultations does them.”* AIS, male, urban CSPS, Zorgho.

However, RDTs were essentially performed by health centre personnel. Community health workers, even though they were involved in the curative management of malaria in patients’ homes, did not perform the tests.

### The logics guiding RDT use: pre-eminence of symptoms

The logic that was guiding RDT use varied greatly from one health professional to another and from one health centre to another. Nevertheless, there was an observed tendency to use RDTs routinely whenever they were available. In most health centres, when they were available, RDTs were performed for most patients, but for different reasons. The first had to do with the acceptance of, and compliance with, national directives regarding the management of suspected malaria:

*“As this is a programme, all we do is follow it; we’re the ones who carry it out. Whatever the decision*-*makers decide, that’s what we do.”* Licensed nurse (IB), male, urban CSPS, Zorgho.

Another had to do with the presence of colleagues or students during consultations; these additional human resources were helpful in managing workload, coping with numbers of patients, and improving test accessibility. In certain situations RDTs were not systematically used, for reasons related to service organization (such as when the RDTs were kept in a location that was not accessible to everyone) or to workload.

It was mainly the symptoms described or presented by patients that guided the use of RDTs: fever/body heat, headache, diarrhoea and vomiting (in children).

*“As for myself, in particular, I don’t systematically do RDTs on every patient who comes in; it depends on the symptoms. If a patient comes in with a cough, I don’t see the need to do an RDT if the patient isn’t reporting any aches and pains, headaches, intermittent fever*—*no symptoms to suggest malaria. And then there are three or four phases of fever that could lead us to suspect malaria; so if I suspect malaria, I do an RDT to be sure, and even in cases of diarrhoea, if it’s accompanied by fever, I do an RDT because malaria can cause diarrhoea.”* IDE, male, urban CSPS B, Kaya.

The most frequent symptom prompting the use of RDT was fever. However, fever was not considered in isolation, as it “*is not necessarily synonymous with malaria*”, as one health professional noted. Respondents referred to other pathologies, such as typhoid fever or otorhinolaryngological infections (e.g., pneumonia, bronchitis) that could cause fever. Conversely, according to the health professionals, “*the absence [of fever] does not exclude malaria*”. Respondents asserted that, according to this logic, the test should be done systematically for all patients, in the spirit of comprehensive patient management.

However, all these logics clearly only applied when the tests were available. Yet this was not always the case, such that health professionals had to fall back on using clinical symptoms to reach their diagnoses. Thus, the new definition of malaria adopted by the health system in 2010 to incorporate RDT results makes sense only if the RDTs are available. Even when they are, however, presumptive diagnoses continue to have a place in patient care.

### Prescribing practices: official directives and empirical practices

The introduction of RDTs modified the official definition of malaria and of the therapeutic management of suspected cases. From then on, according to the national policy,

*“To have an uncomplicated case of malaria, the temperature must be equal to or above 37.5° [Celsius] with a positive RDT result for Plasmodium falciparum*.” IDE, male, urban CSPS B, Kaya.

By the same approach, all drug prescriptions are supposed to be based on evidence of parasite presence obtained through biological diagnosis. However, in the health centres, professionals’ use of RDT results was variable. RDTs had not changed their prescribing practices. This situation was observed more often in Kaya health district, where many patients with negative RDT results continued to receive anti-malarial treatment. The practices were different, however, between urban and rural areas. In urban settings, positive RDT results were never questioned by the health professionals, and those patients received anti-malarial treatment. On the other hand, the same was not true of negative results, which were not always accepted as such, and those patients also often received anti-malarial treatment.

*“Even when the RDT is negative, if the patient presents malaria symptoms, I treat for malaria. Whether the result is negative or positive, when there are certain symptoms (headache, fever, chills, fatigue), I do an RDT but I treat according to the symptoms I see.”* IB, female, urban CSPS A, Kaya.

Of 90 consultations observed in urban CSPS A, RDTs were performed for 70 patients, of which 59 were negative and 11 positive. Of the 59 patients with negative RDTs, 30 were given prescriptions for anti-malarials. In urban CSPS B, of 46 patients seen when RDTs were available, 28 underwent RDTs, of which 11 were positive and 17 negative; five of the negative cases were interpreted and treated as uncomplicated malaria.

In rural areas, only patients with positive RDT results were given anti-malarial treatment. Prescribing anti-malarials for cases with negative RDT results was done less systematically. Anti-malarials were prescribed based on a patient’s symptoms and appeared to be a last resort, after clinical examination of the patient uncovered no other pathology associated with these symptoms.

In Zorgho district, anti-malarials were prescribed in most cases with positive RDT results. Unlike in Kaya, negative RDT results were not routinely diagnosed and treated as cases of uncomplicated malaria in Zorgho district. The observations of consultations, the data in the registers, and even the interviews showed that patients with negative RDTs were rarely given anti-malarial prescriptions. Prescriptions for ACT or quinine remained associated, for the most part, with positive RDTs. Here, the official directives played a significant role in malaria diagnosis and prescriptions. The health professionals referred to the decision algorithm for management of suspected cases of malaria in order to decide on the treatment path based on RDT results. The decision algorithm was a significant reference in rural areas, where no negative RDT (both those observed during the survey and those performed before then) had been diagnosed and treated as malaria.

*“When it [the RDT result] is positive, we prescribe anti*-*malarial treatment, he [the patient] picks it up at the depot, brings it back, and we show him the products. If advice is required, we provide the advice. If it’s negative, we also look to see whether there might be another hidden illness. If we find it, we prescribe something else and he goes to get the product. But I’ve told my people [health professionals] here, if it’s negative, no anti*-*malarials are to be prescribed. We might see it said somewhere that a negative RDT results does not exclude malaria. No! Here, negative RDT means no malaria, look for other symptoms to treat! Here, that’s how it is… That’s in fact what the directives say, that when it’s negative, we don’t give anti*-*malarials… So, when it’s negative we don’t prescribe [anti*-*malarials], the person will come back! Even if it’s not allowed, the person will go for a thick blood smear at Zorgho, where things are more sophisticated. In any case, that’s how it is here.”* IB, male, rural CSPS, Zorgho.

In urban settings, health professionals paid attention to the decision algorithm and tried to comply with it as much as possible. However, they expressed their unease in cases where they were convinced certain patients had malaria even though their RDT results were negative. In those situations, they developed strategies to be able to treat them as malaria cases without giving the impression that they had contravened the official directives. A negative RDT result would be entered in the register as a positive result, or the patient would be given a prescription that was different from what was noted in the register and that would include an anti-malarial treatment. This was observed during a morning consultation conducted by a nurse who, in her interview the previous evening, had stressed the fact that she did not provide anti-malarial treatment when the RDT was negative. She had asserted that, when in doubt, she preferred to request that a thick blood smear be done. Even so, the health professionals considered that such situations were exceptional. In most cases, negative RDTs were not diagnosed and treated as cases of uncomplicated malaria.

Generally speaking, RDTs have not modified health professionals’ prescribing practices. In both districts, the professionals offered several explanations for these coping strategies, the main one being anticipation of complications from an underlying malaria. This logic of anticipating a patient’s status was mentioned in both districts.

*“If the person still has fever but the RDT results continue to be negative, we repeat the clinical exam. If we find nothing else, we might decide to prescribe anti*-*malarial treatment, since we never know whether the persistent fever might be hiding a case of malaria that could very well get worse if not treated in time.”* IDE, female, urban CSPS A, Kaya.

*“Often we prescribe to prevent the worst*-*case scenario, to avoid having the person come back with severe malaria.”* IB, male, urban CSPS, Zorgho.

Thus, treatments were prescribed based on a cautious logic to protect the patient. Some raised doubts about the test’s reliability, while others pointed to certain elements that could influence the results.

*“Because the RDT might often be negative, but if the person was already started on anti*-*malarial treatment, the RDT could show a negative result. Often, even if the treatment was given a week before, we might think it’s malaria, but because treatment was already started, it doesn’t show up as positive.”* AIS, female, urban CSPS B, Kaya.

*“We’re in a region where* Plasmodium falciparum *is predominant, but as I said, there are other types of* Plasmodium *that the RDT doesn’t see, such as* vivax, ovale, etc*. We might also think of those."* IB, male, rural CSPS, Zorgho.

The doubts expressed about the reliability of RDTs explain, in part, why anti-malarials are administered even when RDT results are negative. However, in general, the use of RDT results remained correlated with the symptoms presented or described by the patient, and it was those symptoms that guided the health professionals’ prescribing practices. Fever was the reference symptom. When fever was mentioned or observed, anti-malarials were added to the treatment, even if a patient’s symptoms could be due to other pathologies. In using RDTs, the presumptive diagnosis routinely went hand-in-hand with the biological diagnosis.

### RDTs have certain drawbacks but remain useful

Even though health professionals varied in how they were trained to use them, and even if prescriptions did not always take into account their results, RDTs were assessed positively in both districts. They were perceived as a tool that was easy to use and helpful for managing patient care. Their utility had to do mainly with the support they provided to diagnosis and decision-making.

*“It made our consultations more specialized! Now, with RDTs, we’re sure of the treatment we’re providing, that’s it! There’s a positive diagnosis, and so the treatment becomes very easy. We’re no longer treating in the dark, but instead, with RDT, we know what we’re doing, where we’re going.”* IDE, female, urban CSPS B, Kaya.

Presumptive diagnosis, for a long time the prevalent approach in malaria management that led to haphazard decisions, resulted in erratic care that patients did not necessarily require. According to the health professionals, RDTs facilitated the diagnosis of malaria. They were helpful in establishing a differential diagnosis of symptoms and providing care that was appropriate and more affordable for patients. The fact that RDTs helped to reduce healthcare costs was mentioned more often by health professionals in Zorgho than in Kaya.

*“For example, if you do a test and it’s positive, you know you’re dealing with a case of malaria; and we can also say that RDTs are helpful because everyone…. From a financial standpoint, not everyone in the population has access to a thick blood smear. Even if you think 500 francs isn’t expensive, it isn’t everyone who can have 500 francs to go have a thick blood smear done, so we can say that RDTs, on one hand, are helpful. They reassure the patients.”* IDE, male, urban CSPS, Zorgho.

Moreover, in helping health professionals to make more appropriate and rational prescriptions, RDTs also provided opportunities for them to strengthen their competencies and improve their credibility among patients.

The health professionals mentioned some drawbacks associated with the tool’s use. The lack of gloves for performing the test was mentioned in both districts. This situation exposed them to greater risks, as one respondent explained.

*“We were told we need to wear gloves when doing RDTs, but we were never given any gloves to perform RDTs. There you are, handling blood with your bare hands.”* AIS, male, rural CSPS, Zorgho.

RDTs were also seen by the clinicians as adding to their workload and prolonging the consultation time

*“It takes longer and increases our workload… For example, if you’re alone, you need to write in the ledger, in the booklet, get up to do the RDT, come back and sit around for 10*–*15* *min to wait for the results; it adds to the consultation time.”* IB, female, urban CSPS A, Kaya.

This constraint was felt most keenly in winter, a period of increased activity in health centres. The fact that services for children under 5 years are free exacerbated this perception in the Kaya health centres, which see large numbers of patients, even outside the winter period (when the risk of malaria epidemics is thought to be higher). This led them to adopt coping strategies for the use of RDTs by health professionals: involving workers who are not authorized to prescribe RDTs according to official norms; reorganizing the consultation process during periods of high activity (for example, doing RDTs on all patients in the waiting room before their consultations), and sometimes prescribing RDTs selectively (such as testing only patients with fever).

## Discussion

In this study, the implementation of a public health policy was examined in a natural context [16]. The national scale-up that structured this context made it possible to go beyond the types of reflections often produced from experimental or pilot phases, whose objectives are primarily to evaluate an intervention’s acceptability and feasibility [19]. Generally, in those situations, as in a laboratory, everything is put in place: logistic, human, material, and financial resources in order to ensure an intervention’s success [15]. The scientific process took place in a different situation, in which the tool was incorporated into the health centres’ everyday activities, i.e., into natural working conditions. The advantage of this kind of research is that it makes it possible to assess contextual factors that are known to significantly influence health professionals’ practices and the ways in which they adopt interventions.

### RDT and ACT prescriptions

The introduction of RDTs for malaria is part of a movement to improve health professionals’ diagnostic capacities and promote good therapeutic practices. It involves moving away from presumptive diagnoses in favour of biological diagnoses, such that prescriptions are based on evidence of parasite infection [1]. Clinicians appreciate RDTs as a tool for rapid diagnosis. In the different health centres of the two health districts, the health professionals’ attitudes toward RDTs were definitely heterogeneous, and the logic underlying their use varied. Nevertheless, there was convergence around the need to use this innovation in patient care interactions. With the introduction of these tests, management of suspected cases of malaria was perceived to have become easier and more precise. In this respect, the results of this study corroborated those of other studies, most of which were conducted in experimental contexts [20–22]. The results showed that the tool was appreciated even in natural conditions. The health professionals felt that their competencies were strengthened and their credibility with patients had increased. These feelings stemmed from the fact that using these tests, which offered greater diagnostic precision, helped to undo some entrenched therapeutic habits and thereby reduce the costs of care for patients. Studies in Senegal showed that using RDTs helped lower the overall costs of testing and treatment in cases of fever and, by the same token, avoided “therapeutic overconsumption” [23, 24]. However, while this situation benefited patients in Senegal, it was not so well received by the health centres there, for whom “*malaria is usually the disease that generates a lot of revenue*” [8]. The results of a pilot project conducted in Somalia also showed that the use of RDTs reduced anti-malarial consumption by up to 75 % [25]. In Burkina Faso, RDTs are free for patients in public health centres. The therapeutic management of uncomplicated malaria is based on national norms and protocols derived from WHO recommendations. The drugs used in malaria treatment are ACT. The combination of sulfadoxine-pyrimethanine and quinine tablets is used for intermittent presumptive treatment and for uncomplicated malaria in pregnant women [26]. In CSPSs, the most frequently used treatment remains the combination of artesunate-amodiaquine [12], which is generally prescribed in generic form. ACT is subsidized by the government. There is no financial evidence available on the financial impacts on patients of using RDTs in malaria treatment. Statistical analysis of the CSPS consultation registers to examine the impacts of RDT use on health professionals’ prescribing of anti-malarials for children showed there was no change in ACT prescriptions in the Kaya district. On the other hand, in rural districts of Zorgho, there was a decrease in the trend of prescribing ACT for children [27]. These prescription trends observed in Zorgho corroborate the results of studies conducted in Uganda and Zanzibar, where RDT use led to reductions in anti-malarial prescriptions [28, 29].

The health professionals in Zorgho paid more attention to official directives and took test results into account when prescribing drug treatments, more than the professionals in Kaya health district, who pointed out the financial benefits to patients of using RDTs. They perceived the utility of the tool as a means of bringing some relief to their populations. Other studies in Burkina Faso have shown that contextual factors can explain health professionals’ practices [30]. In this study, there were contextual differences, with free care provided to children under 5 years old on one hand, and a system of user fees on the other, which may partly explain the disparities in health professionals’ practices. In Kaya health district, the costs of consultations, prescriptions and ambulance transport for children under five are covered by a non-governmental organization, whereas in Zorgho there is no financial support aside from that provided by the government. User fee exemption in Kaya has resulted in high use of health centres, especially by parents of children under five, and to some extent has increased workloads [31, 32]. Health professionals there tend to see this increased use of services not so much as a real need for services, but rather as a failure of the free care policy [33]. The impact of context could also apply to the disparity observed between urban and rural settings. Health professionals’ greater compliance with official directives in rural settings, compared with those in urban settings, could in fact be due to the fact that rural health professionals are closer to their populations. Relationships are less anonymous than in the city, which would oblige the professionals to be more conscientious in their work.

### Challenges related to respecting the recommendations on RDT results

The problem does not appear to be related so much to using RDTs as routine work tools, since they are perceived as offering health centres more advantages than disadvantages [34]. Rather, what remains problematic is the buy-in required so that test results will be used to reduce excessive prescribing of drugs and to manage fever cases more efficiently and rapidly [11, 21]. The high cost of ACT currently being used and the fear of seeing resistant strains develop explains the logic underlying the rationalizing of anti-malarial prescriptions [1, 4]. One possible explanation for this discrepancy appears to involve perceptions questioning the quality of the tests and thus their reliability. These reservations are based on health professionals’ own empirical experiences of managing patients. However, rational responses to these reservations are found in RDTs’ objective parameters and certain factors such as parasite density, type of parasite, conditions of transport and preservation, quality of tests used, quantity of blood drawn and self-medication [34, 35]. The respondents in this study expressed frequent doubts regarding the reliability of RDTs.

These factors were assessed in a variety of ways, because, of the diversity of information sources and the means by which health professionals had learned to use the tool. To improve health professionals’ compliance with test results, it is essential that information and knowledge be standardized. Most of the health professionals had not received any formal training, and this need came up repeatedly in their statements. Knowledge was transmitted by means of a cascade (top-down) training strategy implemented over a short period (2–5 days) by people with different interests and profiles. While this approach may have reduced training costs and made it possible to reach many people in a short time, it was seriously limited in terms of effective knowledge dissemination [36]. It entailed the risk of the basic information being diluted, whether by being misinterpreted by those trained at the outset, or being poorly understood by others who were subsequently trained by them [37]. The situation becomes more complicated when the activities are carried out by several actors who, in the course of their activities, provide training that corresponds to their own interests and objectives, but whose content may not always be in line with the objectives of the health authorities [17]. The coping strategies developed by health professionals, with regard to both the handling of RDTs and the use of results, may be seen as a consequence of the lack of uniformity in the dissemination, which allows not only for adjustments, but even for re-interpretations.

Besides the observed heterogeneity of care practices around RDT use, which impaired the proper implementation of the policy, this study highlighted a problem of supply shortages, a recurrent feature of health interventions in Burkina Faso [38, 39] and elsewhere [40]. The poor availability of RDTs and the lack of gloves for performing them were symptomatic of problems faced by the CSPSs.

In the national directives on the use of RDTs, wearing gloves is one of the steps in carrying out the tests. Yet no gloves were provided in the parcels of tests sent to CSPS health professionals, who were left to their own devices in performing the tests. For some, the lack of gloves becomes their justification for not performing RDTs [7]. However, these are the exception, because even though health professionals complained about the lack of gloves, this did not generally prevent them from either performing the test bare-handed or developing strategies to obtain gloves in other ways, such as charging patients for gloves, buying supplies of gloves for themselves, or taking advantage of opportunities presented by the care context. In Kaya, some clinicians used the opportunity provided by the free care funded by a non-government organization to stock up on gloves.

## Limitations of the study

While every effort has been made to ensure the rigorous conduct of this study, nevertheless it has certain limitations. To strengthen the external validity of the results, several other sites could have been studied; but, this was not the fundamental objective of the qualitative methodology used, which was more focused on understanding the phenomena in their complexity. The rigour of the process used [41] helped to strengthen the results and their internal validity. The presence of a social desirability bias [42] that could have influenced respondents to provide positive feedback cannot be excluded, even though the interviews were conducted anonymously to minimize this potential bias.

## Conclusion

The health problem created by malaria has mobilized decision-makers and researchers around issues related to its management. The value of this study is that it was conducted in an uncontrolled context, in contrast to previous studies in Burkina Faso. Health professionals’ adherence to national guidelines is crucial to the successful implementation of a disease management policy, although there are many parameters that influence prescribing behaviours besides health professionals’ command of the technical subject matter. A discrepancy between official norms and practice norms of public actors leads to the question of how health professionals’ practices can be improved [43].

In the absence of other tools to confirm diagnoses, RDTs could be helpful for optimizing care quality and population health status, in that while they do not supplant clinical diagnosis, they can significantly support health professionals’ rapid management of patients. However, in the settings studied here, the prescribing of anti-malarials to patients who had tested negative remained widespread. Efficient use of RDTs will require several improvements in the management of suspected malaria cases: (1) reinforcement of supervision and training practices in order to observe and understand the problems and obstacles that health professionals encounter, as well as any facilitating factors, and to help them to comply with official directives of health programmes; (2) ensure a steady supply of RDTs in health centres; and, (3) update and standardize information and national guidelines provided to health professionals.

## Abbreviations

ACT: artemisinin-based combination therapy
AIS: health outreach worker
CISSE: health information and epidemiological surveillance centres
CSPS: health and social promotion centre
DRD: district distribution depot
ECD: district management team
IB: licensed nurse
IDE: state certified nurse
MCD: district medical officer
RDT: rapid diagnostic test
WHO: World Health Organization
